# Potential health gains for patients with metastatic renal cell carcinoma in daily clinical practice: A real-world cost-effectiveness analysis of sequential first- and second-line treatments

**DOI:** 10.1371/journal.pone.0177364

**Published:** 2017-05-22

**Authors:** S. De Groot, H. M. Blommestein, W. K. Redekop, S. Sleijfer, L. A. L. M. Kiemeney, E. Oosterwijk, C. A. Uyl-de Groot

**Affiliations:** 1 Institute of Health Policy & Management, Erasmus University Rotterdam, Rotterdam, the Netherlands; 2 Department of Medical Oncology and Cancer Genomics Netherlands, Erasmus MC Cancer Institute, Rotterdam, the Netherlands; 3 Department of Urology, Radboud Institute for Molecular Life Sciences, Radboud University Medical Center, Nijmegen, the Netherlands; 4 Department for Health Evidence, Radboud Institute for Health Sciences, Radboud University Medical Center, Nijmegen, the Netherlands; University of Groningen, NETHERLANDS

## Abstract

**Introduction:**

Randomised controlled trials have shown that targeted therapies like sunitinib are effective in metastatic renal cell carcinoma (mRCC). Little is known about the current use of these therapies, and their associated costs and effects in daily clinical practice. We estimated the real-world cost-effectiveness of different treatment strategies comprising one or more sequentially administered drugs.

**Methods:**

Analyses were performed using patient-level data from a Dutch population-based registry including patients diagnosed with primary mRCC from January 2008 to December 2010 (i.e., treated between 2008 and 2013). The full disease course of these patients was estimated using a patient-level simulation model based on regression analyses of the registry data. A healthcare sector perspective was adopted; total costs included healthcare costs related to mRCC. Cost-effectiveness was expressed in cost per life-year and cost per quality-adjusted life-year (QALY) gained. Probabilistic sensitivity analysis was conducted to estimate the overall uncertainty surrounding cost-effectiveness.

**Results:**

In current daily practice, 54% (336/621) of all patients was treated with targeted therapies. Most patients (84%; 282/336) received sunitinib as first-line therapy. Of the patients receiving first-line therapy, 30% (101/336) also received second-line therapy; the majority was treated with everolimus (40%, 40/101) or sorafenib (28%, 28/101). Current treatment practice (including patients not receiving targeted therapy) led to 0.807 QALYs; mean costs were €58,912. This resulted in an additional €105,011 per QALY gained compared to not using targeted therapy at all. Forty-six percent of all patients received no targeted therapy; of these patients, 24% (69/285) was eligible for sunitinib. If these patients were treated with first-line sunitinib, mean QALYs would improve by 0.062–0.076 (where the range reflects the choice of second-line therapy). This improvement is completely driven by the health gain seen amongst patients eligible to receive sunitinib but did not receive it, who gain 0.558–0.684 QALYs from sunitinib. Since additional costs would be €7,072–9,913, incremental costs per QALY gained are €93,107–111,972 compared to current practice.

**Discussion:**

Health can be gained if more treatment-eligible patients receive targeted therapies. Moreover, it will be just as cost-effective to treat these patients with sunitinib as current treatment practice.

## Introduction

Attention for the cost-effectiveness of cancer treatments is swiftly increasing, particularly prompted by the advent of so-called molecularly targeted agents. This class of agents has clearly improved outcomes in several tumor types, but also substantially increased costs.[1] One of the tumor types for which targeted treatments are available is metastatic renal cell carcinoma (mRCC).

In 2008, there were an estimated 88,400 new cases of kidney cancer in Europe.[2] The European mean age-standardised 5-year survival was 60.6%, but substantial differences were seen within European regions.[3] Besides registration artefacts, differences in cancer biology, the use of diagnostic tests and screening, and access to high-quality care might explain the differences in cancer survival.[3]

While previous studies demonstrated a survival benefit from targeted therapies in metastatic renal cell carcinoma,[4–8] a Dutch population-based registry showed that many treatment-eligible patients do not receive sunitinib (or any other targeted therapy) in daily practice.[9] This was also seen in England where one in three patients with mRCC eligible for either sunitinib or pazopanib did not receive the drug.[10] Patient and disease characteristics might play a role in the decision to not prescribe targeted therapy. Another possible reason is that it is not cost-effective to treat these patients.

There is little known about the effect that the potential underuse of targeted therapy in daily clinical practice has on health outcomes and costs. The aim of this study was to estimate the real-world cost-effectiveness of several treatment strategies applied in patients with mRCC comprising one or more sequentially administered drugs.

## Patients and methods

### Study population and data

From the Dutch Cancer Registry, all patients newly diagnosed with mRCC, i.e., metastatic disease at first presentation, from January 2008 until December 2010 in 42 hospitals (both general and academic) in four regions, covering approximately half of The Netherlands, were included in the PERCEPTION registry. In this registry, data on patient characteristics, treatment schemes, treatment endpoints and resource use were retrospectively collected from patient records. Data had been anonymised and de-identified prior to analyses, thus no written informed consent was required. The research protocol was approved by the medical ethics committee of Radboud university medical centre in Nijmegen (CMO Region Arnhem-Nijmegen) in May 2010.

### Model structure and design

A patient-level simulation (PLS) model was developed to model the full disease course of patients newly diagnosed with mRCC. The model comprised entities (i.e., patients), attributes assigned to the entities, and events. Attributes were obtained from patient-level data from the PERCEPTION registry by selecting clinical factors, biochemical and hematologic factors known to impact mRCC outcomes.[11, 12] Events were either second-line treatment or death. The time horizon of the model spanned the patients’ lifetime. The total structure of the model is presented in S1 Fig.

### Parameter estimation and time-to-event

For each patient in the PERCEPTION registry, time from diagnosis of mRCC until the first event (TTE1) (i.e., second-line treatment or death) was calculated. Similarly, the time from start of second-line treatment until the second event (TTE2) (i.e., death) was calculated.

We then compared a range of parametric models to extrapolate the survival data. The fit of different models was assessed systematically by performing Akaike Information Criterion (AIC) and Bayesian Information Criterion (BIC) tests. Additionally, visual inspection was performed by comparing the parametric survival models with the Kaplan-Meier curves.

Clinical factors, biochemical and hematological factors, and the type of targeted treatment were considered for inclusion in the models; TTE1 was also considered as a covariate to estimate TTE2. Backward selection was used to select the attributes for the model; any non-significant attributes (α = 0.10) were excluded from the model one at a time. Forward selection was used to create an alternative model. When two different models were created, AIC and BIC tests were performed, and visual inspection was used to decide on the final model.

Missing data were handled using multiple imputations by chained equations.[13] All statistical analyses were conducted using Stata/SE 13.

### Model calculation

Populations of 621 patients (i.e., the same sample size as the original study population) were repeatedly simulated, one population at a time. Each simulation started with assigning patient and disease characteristics to each patient, based on patient profiles observed in the PERCEPTION registry. That is, random numbers were drawn from predefined distributions for all patient and disease characteristics; similar distributions were used for patients with a similar WHO performance status. For example, the probability of having more than one metastatic site was 64% for patients with a WHO performance status of 0–1, but 73% for patients with a WHO performance status of 2–4. In addition, the previous measurement (e.g., number of metastatic sites before first-line treatment) was taken into account when simulating patient and disease characteristics before second-line treatment.

The TTE1 for each simulated patient was determined by drawing random numbers from two parametric survival models; that is, one model was used to calculate TTE1 until 12 months while a second model was used to calculate TTE1 after 12 months. Two models were used since the probability of an event (i.e., second-line treatment or death) from 12 months onwards was underestimated when only one single model was used. The type of event (i.e., second-line treatment or death) was determined using a separate model. The TTE2 was calculated in a similar manner.

If a patient’s modelled time to an event was longer than the remaining life expectancy based on national vital statistics data,[14] we used national vital statistics data to estimate TTE1 and TTE2 because it is not plausible for someone with mRCC to have a longer than average life expectancy.

### Treatment scenarios

**Fig 1:**
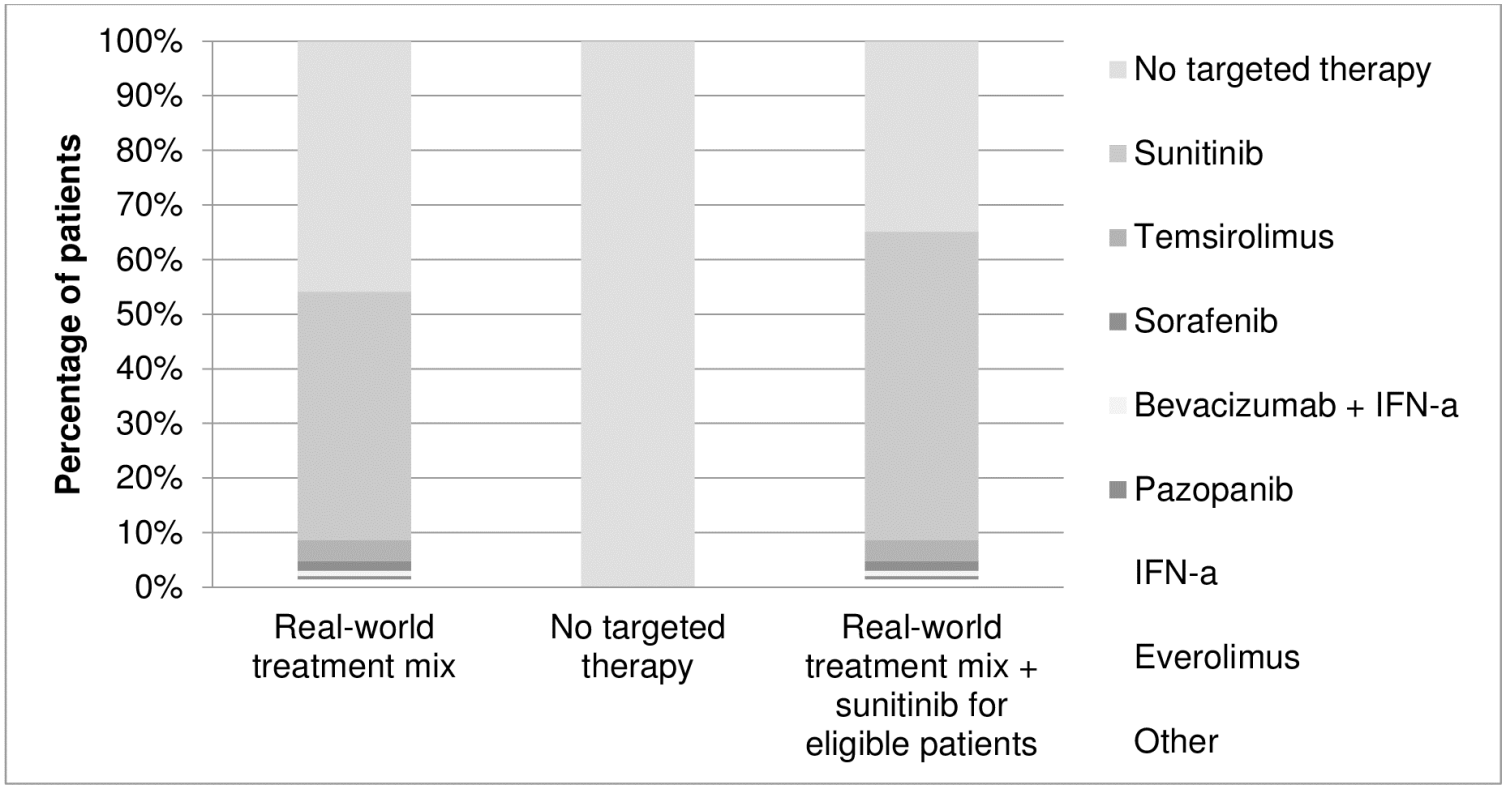
First-line therapies in the various treatment scenarios.

In the base-case scenario, patients were treated as they were in the real world (Fig 1). A multinomial logistic regression model based on patient-level data from the PERCEPTION registry was used to predict the type of treatment in both first- and second-line, using values for WHO performance status, haemoglobin, corrected serum calcium and lactate dehydrogenase (i.e., 4/5 Memorial Sloan Kettering Cancer Center (MSKCC) criteria).[15]

Alternative scenarios included no targeted therapy for all patients, or treating all patients just as they were in reality, except for one difference, namely that first-line sunitinib followed by sorafenib, everolimus or another second-line treatment was given to patients who did not receive any targeted treatment even though they fulfilled the SUTENT trial eligibility criteria (Fig 1). A patient was classified as fulfilling SUTENT trial eligibility criteria if he had a clear-cell subtype, a WHO performance status of 0 or 1 and no brain metastases.[16]

The potential health outcomes and costs of all treatment scenarios were calculated by running the model for 621 simulated patients.

### Health outcomes

Health outcomes were estimated in terms of life-years (LYs) and quality-adjusted life-years (QALYs). QALYs were calculated by weighting LYs for the quality of life during these years using utility weights derived from the published literature (S1 Table).[17–19]

### Resource use and costs

This cost-effectiveness analysis (CEA) was conducted from a health care sector perspective, but only included health care costs related to mRCC, i.e., drug costs as well as resource utilisation costs, such as hospitalisations, outpatient visits and medical imaging services. Hospitalisations due to adverse events were included while other types of costs due to adverse events, such as concomitant medications, were not. The calculation of drug costs and resource utilisation costs is described in S1 Text.

Costs were reported in Euro 2014. Wherever necessary, costs were adjusted to 2014 using the general price index derived from Statistics Netherlands.[20] Costs and effects were discounted at 4% and 1.5%, respectively.[21]

### Model validation

The model was internally validated by comparing patient characteristics and OS observed in the PERCEPTION registry to patient characteristics and health outcomes according to the model.[22] Health outcomes from the model were presented as the mean of 1000 iterations with a 95% confidence interval (C.I.) using the standard deviation of 1000 iterations as standard error of the mean. The model’s internal validity was assessed by evaluating whether OS observed in the PERCEPTION registry fell within the 95% C.I. of the OS according to the base-case scenario of the model.

### Sensitivity analysis

Univariate sensitivity analyses were performed to examine the impact of alternative input parameters on the incremental cost-effectiveness ratios (ICERs). Probabilistic sensitivity analysis (PSA) was conducted to examine the impact of the joint uncertainty regarding all input parameters on the results.

## Results

### Study population and treatment

714 patients in the Dutch Cancer Registry fulfilled the inclusion criteria. 39 patients were excluded (S2 Fig), and an additional 30 patients were lost to follow-up. Complete follow-up up to three years after diagnosis was available for 645 patients. Twenty-four of these patients received a metastasectomy (combined with a nephrectomy) with a possible curative intention, making targeted treatment redundant. These patients were therefore excluded from the analyses.

Patient characteristics are shown in Table 1, along with the characteristics after multiple imputation. The distribution of patients according to the MSKCC risk score showed a high proportion of patients (55%) with a poor prognosis. 42% of the patients had an intermediate prognosis. Since all patients presented with metastatic disease, very few patients (3%) had a favourable prognosis (e.g., 86% of the patients had a time from diagnosis to treatment, which is one of the MSKCC criteria, of less than one year).

**Table 1:** Patient and disease characteristics before start of first- and second-line treatment.

	*First-line*		*Second-line*	
	Real world-data (N = 621)	Average of imputed datasets (N = 621)	Real world-data (N = 101)	Average of imputed datasets (N = 101)
Sex—N (%)				
Female	213 (34%)	213 (34%)	27 (27%)	27 (27%)
Male	408 (66%)	408 (66%)	74 (73%)	74 (73%)
Median age—yr (range)	66 (23–93)	66 (23–93)	62 (23–79)	62 (23–79)
Histology—N (%)				
Clear cell	354 (57%)	354 (57%)	69 (68%)	69 (68%)
Other *	267 (43%)	267 (43%)	32 (32%)	32 (32%)
WHO performance status—N (%)				
0–1	204 (33%)	430 (69%)	34 (34%)	73 (72%)
2–4	61 (10%)	191 (31%)	9 (9%)	28 (28%)
Missing	356 (57%)		58 (57%)	
Site of metastasis—N (%)				
one	195 (31%)	206 (33%)	19 (19%)	19 (19%)
more than one	398 (64%)	415 (67%)	82 (81%)	82 (81%)
Missing	28 (5%)		0 (0%)	
Liver metastasis—N (%)				
no	487 (78%)	509 (82%)	74 (73%)	74 (73%)
yes	106 (17%)	112 (18%)	27 (27%)	27 (27%)
Missing	28 (5%)		0 (0%)	
Lung metastasis—N (%)				
no	163 (26%)	173 (28%)	21 (21%)	21 (21%)
yes	430 (69%)	448 (72%)	80 (79%)	80 (79%)
Missing	28 (5%)		0 (0%)	
Bone metastasis—N (%)				
no	375 (60%)	393 (63%)	58 (57%)	58 (57%)
yes	218 (35%)	228 (37%)	43 (43%)	43 (43%)
Missing	28 (5%)		0 (0%)	
Brain metastasis—N (%)				
no	546 (88%)	571 (92%)	92 (91%)	92 (91%)
yes	47 (8%)	50 (8%)	9 (9%)	9 (9%)
Missing	28 (5%)		0 (0%)	
Prior nephrectomy—N (%)				
no	452 (73%)	453 (73%)	43 (43%)	43 (43%)
yes	168 (27%)	168 (27%)	58 (57%)	58 (57%)
Missing	1 (0%)		0 (0.0%)	
Haemoglobin—N (%)				
normal	171 (28%)	205 (33%)	20 (20%)	20 (20%)
< LLN	347 (56%)	416 (67%)	78 (77%)	81 (80%)
Missing	103 (17%)		3 (3%)	
Neutrophil count—N (%)				
normal	203 (33%)	383 (62%)	67 (66%)	88 (87%)
> ULN	108 (17%)	238 (38%)	10 (10%)	13 (13%)
Missing	310 (50%)		24 (24%)	
Platelet count—N (%)				
normal	358 (58%)	452 (73%)	66 (65%)	70 (69%)
> ULN	140 (23%)	169 (27%)	29 (29%)	31 (31%)
Missing	123 (20%)		6 (6%)	
Albumin—N (%)				
normal	247 (40%)	391 (63%)	51 (51%)	75 (74%)
< LLN	136 (22%)	230 (37%)	18 (18%)	26 (26%)
Missing	238 (38%)		32 (32%)	
Corrected serum calcium—N (%)				
normal	243 (39%)	421 (68%)	45 (45%)	72 (71%)
> ULN	116 (19%)	200 (32%)	18 (18%)	29 (29%)
Missing	262 (42%)		38 (38%)	
Alkaline phosphatase—N (%)				
normal	324 (52%)	432 (70%)	65 (64%)	74 (73%)
> ULN	139 (22%)	189 (30%)	24 (24%)	27 (27%)
Missing	158 (25%)		13 (13%)	
Lactate dehydrogenase—N (%)				
normal	277 (45%)	372 (60%)	63 (62%)	71 (70%)
> 1.5 times ULN	174 (28%)	249 (40%)	28 (28%)	30 (30%)
Missing	170 (27%)		10 (10%)	

Abbreviations: LLN, lower limit of normal; ULN, upper limit of normal.

* mRCC was clinically established without histopathological confirmation in 17% of patients and mRCC was classified as not otherwise specified without further subtyping in 13% of patients. It is likely that a substantial proportion of these patients had a clear cell subtype.

Fifty-four percent (336/621) of all patients was treated with targeted therapies. Of these patients, 84% (282/336) received sunitinib as first-line therapy. Other first-line treatments given were temsirolimus (7%, 24/336) and sorafenib (3%, 11/336). 101 patients also received a second-line therapy; the majority was treated with everolimus (40%, 40/101) or sorafenib (28%, 28/101). Median overall survival (OS) of patients treated with targeted therapies was 12.6 months (95% C.I. 10.5–14.8).

Almost half (46%, 285/621) of all patients did not receive any targeted therapy. Of these 285 patients, 69 patients (24%) fulfilled the SUTENT trial eligibility criteria. Most patients (76%) did not fulfill the SUTENT trial eligibility criteria; 168 patients (78%) did not have a clear-cell subtype, 46 patients (21%) did not have a WHO performance status of 0 or 1 and 2 patients (1%) had brain metastases. Median OS of patients not treated with targeted therapies was 2.6 months (95% C.I. 2.1–3.5); 10.6 months (95% C.I. 3.8–18.6) for patients fulfilling the SUTENT trial eligibility criteria and 1.9 months (95% C.I. 1.6–2.6) for patients not fulfilling the SUTENT trial eligibility criteria.

Table 2 shows the final models with their covariates (e.g., patient and disease characteristics) and corresponding coefficients to estimate TTE1, the type of event after TTE1 and TTE2. For example, a WHO performance status of 2–4 means a shorter TTE1.

**Table 2:** Covariates and corresponding coefficients of the survival models and logistic regression model.

	Time to event 1 (first 12 months)	Time to event 1 (> 12 months)	Type of event 1*	Time to event 2
**Model type**	Loglogistic	Exponential	Logistic	Weibull
				
**Covariate**	**Coefficient (s.e.)**	**Coefficient (s.e.)**	**Coefficient (s.e.)**	**Coefficient (s.e.)**
**Constant**	1.060 (0.393)	5.227 (0.663)	-1.778 (0.790)	2.804 (0.249)
**Age (yr)**	0.012 (0.005)	-0.027 (0.009)	0.037 (0.013)	
**Sex (male vs. female)**				-0.382 (0.185)
**Histology (non-clear cell vs. clear cell)**	-0.229 (0.112)			
**Prior nephrectomy (yes vs. no)**	0.783 (0.130)			
**Number of metastatic sites (more than 1 vs. 1)**	-0.306 (0.120)			-0.387 (0.199)
**WHO performance status (2–4 vs. 0–1)**	-0.585 (0.136)			
**Liver metastases (yes vs. no)**	-0.459 (0.137)			0.632 (0.164)
**Bone metastases (yes vs. no)**	0.277 (0.113)	-0.330 (0.170)		-0.421 (0.150)
**Brain metastases (yes vs. no)**				-0.657 (0.238)
**Thrombocytes (>ULN vs. normal)**			0.587 (0.320)	-0.360 (0.183)
**Neutrophil count (>ULN vs. normal)**	-0.258 (0.136)			
**Albumin (<LLN vs. normal)**	-0.290 (0.137)		1.074 (0.381)	-0.388 (0.196)
**Alkaline phosphatase (>ULN vs. normal)**	-0.269 (0.129)			
**First-line sunitinib (vs. no targeted therapy)**	0.915 (0.124)	-0.602 (0.201)		
**First-line temsirolimus (vs. no targeted therapy)**	0.580 (0.244)	-1.529 (0.611)		
**First-line other (vs. no targeted therapy)**	0.992 (0.273)	0.339 (0.375)		
**Second-line everolimus (vs. sorafenib)**				-0.143 (0.191)
**Second-line other (vs. sorafenib)**				-0.388 (0.181)
**TTE 1 (TTE 1 > 12 months vs. TTE 1 < = 12 months)**			-0.641 (0.270)	0.537 (0.147)
**Shape parameter**	0.669 (0.028)			1.625 (0.137)

NOTE. Lung metastases, heamaglobin, corrected serum calcium and lactate dehydrogenase were considered for inclusion in the survival models and logistic regression model, but excluded through backward and/or forward selection.

Abbreviations: LLN, lower limit of normal; ULN, upper limit of normal; TTE, time to event.

*0 = second-line therapy/ 1 = death.

### Internal validation

Observed data from the PERCEPTION registry showed a median OS of 7.3 months (0.6 LYs) (95% C.I. 6.3–8.4) for the total population (in which 54% of the patients received a targeted therapy). Median OS in the model was 7.0 months (0.6 LYs) (95% C.I. 5.7–8.3) if patients were treated as they were in the real world (i.e., base-case scenario). The OS derived from the PERCEPTION registry fell within the 95% C.I. of the outcome of the model. Additionally, the observed Kaplan Meier curves (TTE1, TTE2 and OS) were closely followed by the survival curves derived from the model (S3–S5 Figs).

### Effectiveness

The model yielded an estimated mean survival of 1.2 LYs (14.4 months) if patients were treated as they were in the real world (in which 54% of the patients received a targeted therapy). If all treatment-eligible patients would be treated with first-line sunitinib followed by sorafenib, everolimus or another second-line treatment (if they did not die after first-line treatment), mean survival would increase to 1.3 LYs (15.6 months). If none of the patients were to be treated with any targeted therapy, mean survival would decrease to 0.9 LYs (10.8 months) (Table 3).

**Table 3:** Summary of the cost-effectiveness results.

	Real-world treatment mix	No targeted therapy	Real-world treatment mix + sunitinib for eligible patients (followed by sorafenib)	Real-world treatment mix + sunitinib for eligible patients (followed by everolimus)	Real-world treatment mix + sunitinib for eligible patients (followed by other)
**Per strategy**					
**Time to event 1 (years)—mean (95% CI)**	1.1 (0.9–1.3)	0.9 (0.6–1.1)	1.1 (1.0–1.3)	1.1 (1.0–1.3)	1.1 (1.0–1.3)
Time to event 2 (years)—mean (95% CI)*	0.7 (0.5–0.9)	NA	0.9 (0.6–1.2)	0.8 (0.5–1.0)	0.6 (0.4–0.8)
					
**LYs—mean (95% CI)**	1.2 (1.0–1.4)	0.9 (0.6–1.1)	1.3 (1.1–1.5)	1.3 (1.1–1.5)	1.3 (1.1–1.5)
**QALYs—mean (95% CI)**	0.807 (0.647–0.966)	0.576 (0.403–0.749)	0.883 (0.719–1.046)	0.868 (0.709–1.027)	0.841 (0.687–0.996)
**Total costs—mean (95% CI)**	€58,912 (€48,393–€69,431)	€34,733 (€23,164–€46,301)	€65,984 (€55,009–€76,959)	€65,825 (€54,661–€76,989)	€65,062 (€54,106–€76,018)
					
**Compared to the real-world treatment mix**					
**LYs gained—mean (95% CI)**	NA	-0.4 (-0.6–-0.1)	0.1 (-0.1–0.3)	0.1 (-0.1–0.3)	0.1 (-0.1–0.2)
**QALYs gained—mean (95% CI)**	NA	-0.230 (-0.390–-0.070)	0.076 (-0.056–0.208)	0.062 (-0.068–0.191)	0.035 (-0.094–0.163)
**Incremental costs—mean (95% CI)**	NA	-€24,179 (-€34,856–-€13,502)	€7,072 (-€2,070–€16,214)	€6,913 (-€2,252–€16,079)	€6,150 (-€2,867–€15,167)
**Cost/ LYG**	NA	NR	€60,716	€73,485	€117,814
**Cost/ QALY**	NA	NR	€93,107	€111,972	€177,226
					
**Compared to no targeted therapy**					
**LYs gained—mean (95% CI)**	0.4 (0.1–0.6)	NA	0.5 (0.2–0.7)	0.4 (0.2–0.7)	0.4 (0.1–0.7)
**QALYs gained—mean (95% CI)**	0.230 (0.070–0.390)	NA	0.306 (0.126–0.486)	0.292 (0.114–0.470)	0.265 (0.088–0.442)
**Incremental costs—mean (95% CI)**	€24,179 (€13,502–€34,856)	NA	€31,251 (€18,848–€43,654)	€31,093 (€18,391–€43,794)	€30,329 (€17,734–€42,924)
**Cost/ LYG**	€69,068	NA	€66,983	€70,003	€75,393
**Cost/ QALY**	€105,011	NA	€102,058	€106,483	€114,469

Note. Results are discounted (benefits 1.5% and costs 4%).

Abbreviations: LYs, life years; QALYs, quality-adjusted life years; LYG, life years gained; CI, confidence interval; NA, not applicable; NR, not reported.

* Time to event 2 is only relevant for patients who received a second-line therapy.

If patients were treated as they were in the real world, mean QALYs are 0.807. If all treatment-eligible patients would be treated with first-line sunitinib followed by sorafenib, everolimus or another second-line treatment, mean QALYs would increase to 0.883, 0.868 or 0.841 respectively. If none of the patients were to be treated with any targeted therapy, mean QALYs would decrease to 0.576 (Table 3).

### Costs

Mean total costs per treatment strategy are presented in Table 3. Mean total costs per patient amount to €58,912 if patients were treated as they were in the real world. If all treatment-eligible patients were to be treated with first-line sunitinib followed by sorafenib, everolimus or another second-line treatment, mean total costs would increase to €65,984, €65,825 or €65,062 respectively. If none of the patients would be treated with any targeted therapy, mean total costs would decrease to €34,733.

### Cost-effectiveness

Compared to a scenario in which none of the patients receives a targeted therapy, the real-world treatment mix results in a QALY gain of 0.230 and a cost increase of €24,179. Thus, an additional €105,011 per QALY gained is spent compared to the scenario of not using targeted therapy.

Compared to the real-world treatment mix, first-line sunitinib followed by sorafenib, everolimus or another second-line treatment leads to a QALY gain of 0.076, 0.062 and 0.035 at the population level, respectively. When combined with the corresponding incremental costs (i.e., €7,072, €6,913 and €6,150), the incremental costs per QALY gained are €93,107, €111,972 and 177,226, respectively (Table 3). Note that the health gains are achieved by changing the treatment of a relatively small group of patients representing 11% of the population. These are patients who were eligible to receive sunitinib but did not receive it in real life; in this group, sunitinib leads to a gain of 0.684, 0.558 or 0.315 QALYs per patient.

### Sensitivity analyses

**Fig 2:**
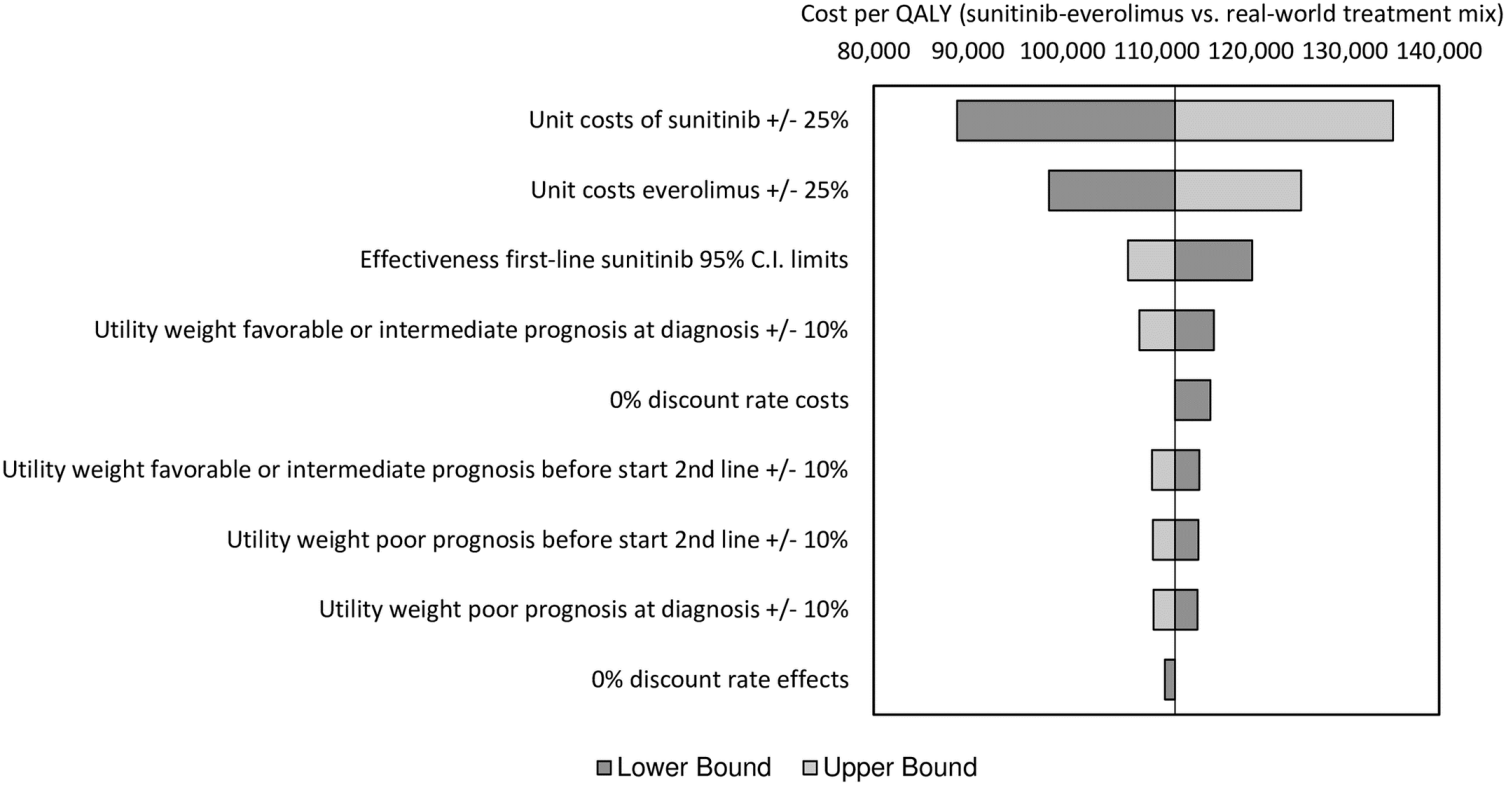
Results of the univariate sensitivity analyses.

The tornado diagram (Fig 2) shows the variability in the ICER of sunitinib followed by everolimus compared to the real-world treatment mix as a consequence of changes in the values of various input parameters. Varying the unit costs of first-line sunitinib has the highest impact on the ICER.

**Fig 3:**
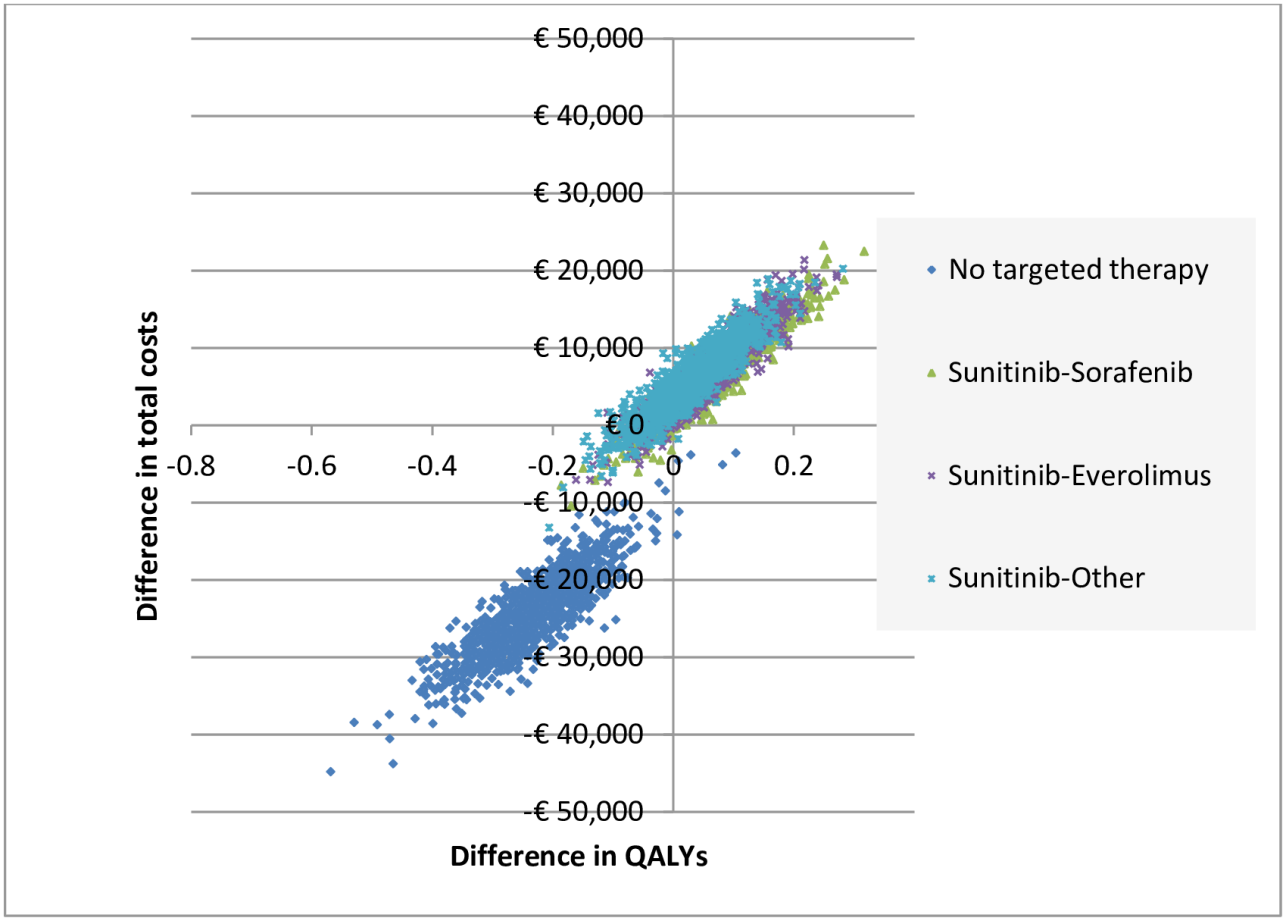
Cost-effectiveness plane for various treatment scenarios versus real-world treatment mix.

Fig 3 shows the uncertainty around the total costs and QALYs as obtained from the PSA. For sunitinib followed by sorafenib, everolimus or another second-line treatment, 88.3%, 82.3% and 72.1% of all simulations fell in the north-east quadrant indicating more QALYs and higher costs compared to the real-world treatment mix. For the scenario in which none of the patients received a targeted therapy, 99.4% of all simulations fell in the south-west quadrant indicating less QALYs and lower costs.

**Fig 4:**
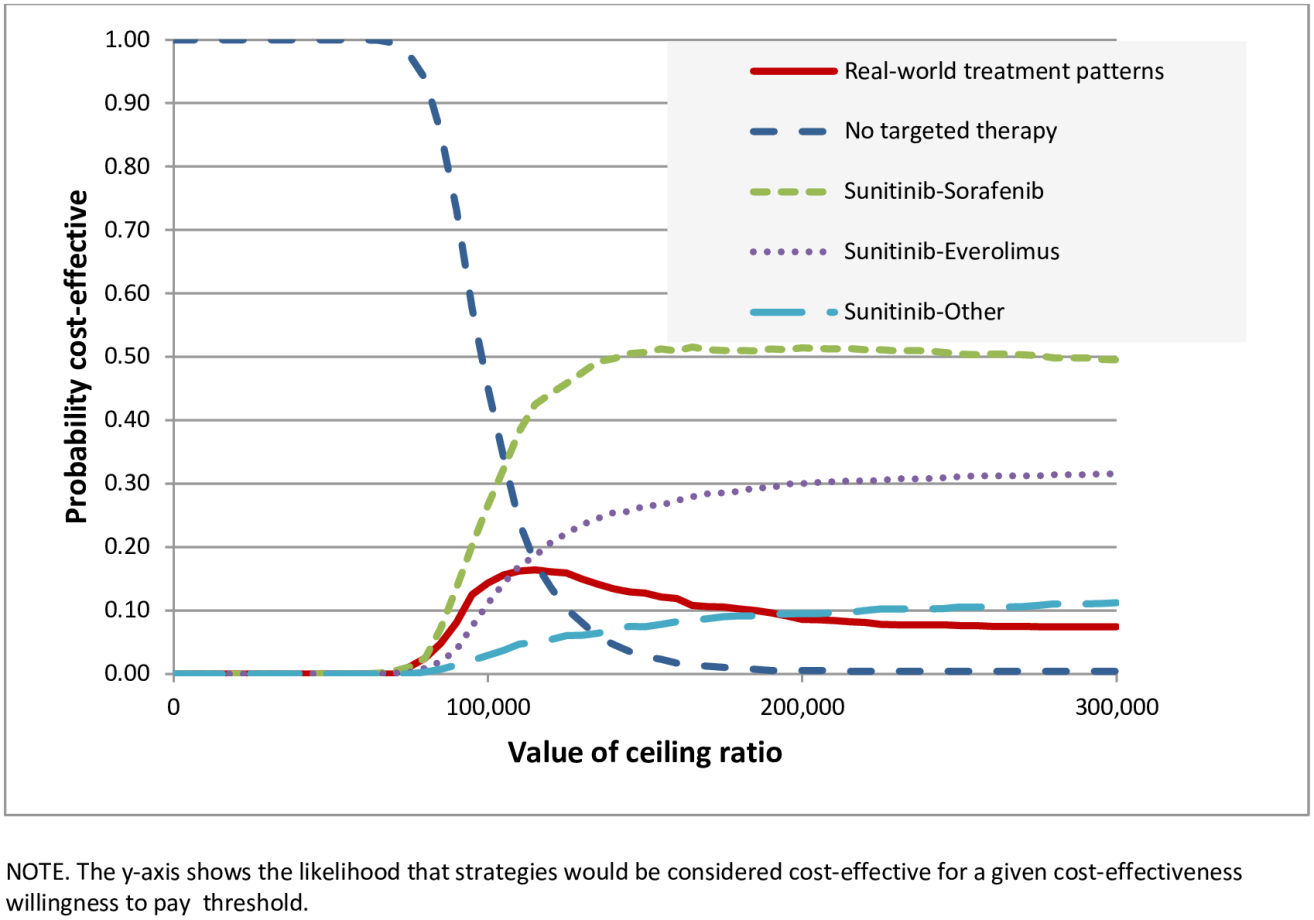
Cost-effectiveness acceptability curves representing the probability that each treatment strategy is cost-effective for a given maximum willingness-to-pay threshold per QALY gained.

Cost-effectiveness acceptability curves are presented in Fig 4, showing the likelihood that treatment strategies would be cost-effective at a given willingness-to-pay threshold. Treating according to the real-world treatment mix never attains more than 16% of simulations. Treating all patients with sunitinib followed by sorafenib or everolimus would be favoured; these scenarios have a probability of 34% and 15%, respectively, of being cost-effective at a willingness-to-pay threshold of €106,000. Not treating any patient with a targeted therapy would be preferred at willingness-to-pay thresholds below €106,000, but this scenario results in health loss.

## Discussion

To the best of our knowledge, this is the first study that models the full disease course of patients with mRCC using real-world data. We found that real-world treatment of mRCC patients yields a QALY gain of 0.230 with incremental costs of €24,179 compared to a scenario in which none of the patients would receive a targeted therapy. Thus, we currently pay €105,011 per QALY gained. However, only 54% of the patients in our study population received a targeted therapy and this raises the question about what the potential impact would be if all treatment-eligible patients were to receive targeted therapy. Compared to real-world treatment, health can be gained if all eligible patients were to be treated with first-line sunitinib followed by sorafenib or everolimus. The costs to gain health by treating all eligible patients with these treatment strategies, i.e., €93,107 and €111,972 per QALY gained, respectively, are similar to the current costs per QALY gained. These costs include the costs of both first- and second-line therapy.

The proportion of patients not being treated in this series is high at 46% (285/621). However, not all these patients were eligible for targeted therapy. We found that one in four patients (69/268; 26%) fulfilling SUTENT trial eligibility criteria did not receive any targeted therapy. Also in England, one in three patients with mRCC eligible for either sunitinib or pazopanib did not receive the drug.[10] Previous analyses indicated that patients aged 65+ years were less likely to receive targeted therapy than younger patients after adjustment for other factors.[9] However, the exact causes underlying the remarkably high proportion of non-treated patients deserve further study. Importantly, all drugs studied in this project were available in the Netherlands during the study period without any limitations for patients or prescribers, so this could not explain why many eligible patients in Dutch daily clinical practice did not receive targeted therapy.

Multiple economic evaluations of targeted therapies in mRCC have been published using data from RCTs, two of which examined the cost-effectiveness of sunitinib.[23–27] Several explanations exist for differences regarding the cost-effectiveness of sunitinib between our study and these two studies (by Remák et al. and Benedict et al.).[23, 25] To start with, we used real-world data (using the PERCEPTION registry) while the other studies used data from key clinical trials. In addition, we looked at all patients with mRCC (at the initial presentation), while the other two studies just studied one subgroup (i.e., sunitinib-eligible patients).

Some limitations to the data and methods deserve mentioning. First, since data from all patients newly diagnosed with mRCC at the initial presentation in 42 hospitals (both general and academic) were collected, it is likely that these patients are representative of average patients with mRCC at initial diagnosis and the average treatment in The Netherlands. However, these patients account for only 40%-70% of the total mRCC population.[28] This total population also includes patients who initially presented with non-metastatic disease and later developed distant metastases. It is likely that a lot more of these patients are being treated; a CEA based on these patients will likely yield different results.

Second, the model was populated using data from primary mRCC patients diagnosed between January 2008 and December 2010 (i.e., treated between 2008 and 2013), whereas new treatments have become available since then. The PERCEPTION registry also included a cohort of (m)RCC patients diagnosed between January 2011 and June 2013, but since inclusion criteria differed, patients were identified differently and the duration of follow-up varied, it was not feasible to include these patients in the current study. Nevertheless, we did not observe an increase in the proportion of patients receiving targeted therapies in this population.[9] Therefore, we believe that the conclusion of this study still applies, and health can be gained if more treatment-eligible patients receive targeted therapies.

Third, the alternative scenarios included in this model assume that all treatment-eligible patients can be treated with a certain targeted therapy. This assumption may overestimate the number of eligible patients since some of these patients eligible on the basis of the data captured in the PERCEPTION registry, may not actually be eligible because of poor organ function or comorbidities.

Fourth, treatment costs were overestimated somewhat since we did not adjust for dose reductions. However, the effect on the incremental costs and ICERs will be minimal since the treatment costs of 54% of the patients in the base-case real-world scenario were also overestimated somewhat. Another limitation of this study is the amount of missing data in baseline characteristics. Multiple imputations by chained equations were conducted to overcome this problem.[13] This method ensures that all patients are included in the analysis but simultaneously guarantees that the uncertainties from missing data are retained.

In conclusion, RCTs have shown that targeted therapies like sunitinib are effective in mRCC treatment. RCT-based cost-effectiveness analyses with a lifetime time horizon provide important information about the cost-effectiveness of these therapies. However, these analyses are limited in scope, since they are conducted in a selected population. A full disease model and real-world data as presented here are essential in estimating cost-effectiveness ratios that are externally valid. We found that one in four patients eligible for sunitinib did not receive it. It is difficult to state with certainty why these patients did not receive sunitinib. One possible reason is a limited health gain from treatment with sunitinib, but this reasoning seems unlikely since we estimated that its use may add 0.684 QALYs (or eight months in perfect health) to individual patients. Another possible reason is that it is not cost-effective to treat these patients. However, we found that it is just as cost-effective to treat these patients with sunitinib as current treatment practice.

## Supporting information

S1 Text Calculation of drug costs and resource utilisation costs. (DOCX)

S1 Table Utility weights. (DOCX)

S1 Fig Model structure. (TIF)

S2 Fig Patient enrollment. (TIF)

S3 Fig Comparison of time to event 1 (i.e., time from diagnosis to second-line treatment or death) between original and simulated data. (TIF)

S4 Fig Comparison of time to event 2 (i.e., time from start of second-line treatment to death) between original and simulated data. (TIF)

S5 Fig Comparison of overall survival (i.e., time from diagnosis to death) between original and simulated data. (TIF)
