# Supplementary material for: Potential health gains for patients with metastatic renal cell carcinoma in daily clinical practice: A real-world cost-effectiveness analysis of sequential first- and second-line treatments
Source: PLoS One. 2017 May 22;12(5):e0177364. doi: 10.1371/journal.pone.0177364 (PMC5439671; doi:10.1371/journal.pone.0177364)
Supplement: S1 Text — (DOCX) [file pone.0177364.s001.docx]

**S1 Text. Calculation of drug costs and resource utilisation costs**

Drug costs were calculated by multiplying monthly costs of a therapy [29] by the time to an event (Table A). The PERCEPTION registry showed that, if patients did not undergo a cytoreductive nephrectomy prior to drug treatment, they started drug treatment 1.4 months (s.d. 1.7, n=219) after diagnosis. If patients underwent cytoreductive nephrectomy prior to drug treatment, they started drug treatment 8.6 months (s.d. 9.8 months, n=116) after diagnosis. Therefore, in the model we assumed patients started drug treatment one month after diagnosis, except for patients who underwent a nephrectomy prior to drug treatment; we assumed that these patients started drug treatment nine months after diagnosis.

We also assumed patients discontinued drug treatment either one month before start of second-line treatment or three months before death. These assumptions are also based on the PERCEPTION registry, but since the date of discontinuation was often lacking in the PERCEPTION registry, it was not possible to calculate the time between discontinuation and either start of second-line treatment or death for all patients in the registry. This assumption was therefore verified by a clinical expert who participated in the registry.

A maximum first-line treatment duration of 41 months, and a maximum second-line treatment duration of 34.1 months was assumed based on the results from other studies.[30,31]

Monthly resource use per treatment strategy was derived from patient-level data (Table B). Total costs were calculated by multiplying monthly resource use by unit costs,[32,33] and the time to an event.

**Table A. Drug costs**

| **Drug** | **Dose and frequency** | **Costs**  **(Euros)** | **Costs per month**  **(Euros)** | **Source** |
| --- | --- | --- | --- | --- |
| Sunitinib | 50 mg daily for 4 weeks, followed by 2-week rest period | 184 per 50 mg | 3,727 | 29 |
| Temsirolimus | 25mg once per week | 928 per dose | 4,030 | 29 |
| Sorafenib | 400 mg twice daily | 35 per 200 mg | 4,243 | 29 |
| Everolimus | 10 mg daily | 129 per 10 mg | 3,931 | 29 |

**Table B. Unit costs and resource use per treatment strategy per month**

| **Resources** | **Unit costs**  **Euros** | **No targeted therapy**  **N=215**  **Mean (s.e.)** | **End of life (<1 mo)**  **N=72**  **Mean (s.e.)** | **First-line sunitinib**  **N=281**  **Mean (s.e.)** | **First-line temsirolimus**  **N=23**  **Mean (s.e.)** | **Second-line sorafenib**  **N=28**  **Mean (s.e.)** | **Second-line everolimus**  **N=40**  **Mean (s.e.)** |
| --- | --- | --- | --- | --- | --- | --- | --- |
| Inpatient days - non-ic | 530 | 5.1 (0.5) | 18.4 (1.3) | 2.7 (0.2) | 4.4 (1.0 ) | 1.5 (0.6) | 2.6 (0.5) |
| Inpatient days - ic | 2,401 | 0.1 (0.0) | 1.2 (0.5) | 0.0 (0.0) | 0.1 (0.1) | 0.0 (0.0) | 0.0 (0.0) |
| Outpatient visits | 94 | 1.4 (0.1) | 1.9 (0.3) | 1.9 (0.1) | 2.4 (0.3) | 1.4 (0.2) | 2.2 (0.3) |
| Day care treatments | 276 | 0.1 (0.0) | 0.1 (0.0) | 0.2 (0.0) | 2.0 (0.2) | 0.3 (0.1) | 0.3 (0.1) |
| Emergency room visits | 166 | 0.2 (0.0) | 0.8 (0.1) | 0.1 (0.0) | 0.2 (0.1) | 0.1 (0.0) | 0.2 (0.0) |
| Laboratory | 5 | 6.2 (1.0) | 29.0 (5.7) | 4.2 (0.4) | 5.9 (1.0) | 2.4 (0.6) | 4.2 (0.7) |
| X-ray | 53 | 0.8 (0.1) | 4.1 (0.7) | 0.6 (0.1) | 1.4 (0.3) | 0.5 (0.2) | 0.8 (0.2) |
| CT-scan | 189 | 0.5 (0.0) | 1.6 ( 0.3) | 0.5 (0.0) | 0.7 (0.1) | 0.4 (0.1) | 0.5 (0.1) |
| MRI | 281 | 0.1 (0.0) | 0.2 ( 0.1) | 0.1 (0.0) | 0.1 (0.0) | 0.0 (0.0) | 0.0 (0.0) |
| Ultrasound | 88 | 0.2 (0.0) | 0.9 ( 0.2) | 0.2 (0.0) | 0.2 (0.0) | 0.1 (0.1) | 0.1 (0.0) |
| PET-CT | 1,163 | 0.0 (0.0) | 0.0 ( 0.0) | 0.0 (0.0) | 0.0 (0.0) | 0.0 (0.0) | 0.0 (0.0) |
| Skeletal scintigraphy | 244 | 0.1 (0.0) | 0.1 ( 0.1) | 0.0 (0.0) | 0.0 (0.0) | 0.0 (0.0) | 0.0 (0.0) |
| Heart scintigraphy (MUGA) | 314 | 0.0 (0.0) | 0.1 ( 0.1) | 0.0 (0.0) | 0.0 (0.0) | 0.0 (0.0) | 0.0 (0.0) |
| Electrocardiogram | 16 | 0.0 (0.0) | 0.2 ( 0.1) | 0.1 (0.0) | 0.1 (0.0) | 0.0 (0.0) | 0.1 (0.0) |

NOTE. Unit costs of inpatient days, day treatments and outpatient visits were based on detailed microcosting studies.[32] Resource use related to imaging services was valued using the tariffs as issued by the Dutch Healthcare authority.[33]

**References**

29. Dutch reimbursement prices (Z-index) [in Dutch: Officiële vergoedingsprijzen (Z-index)]. 2015.

30. Motzer RJ, Hutson TE, Tomczak P, Michaelson MD, Bukowski RM, Oudard S, et al. Overall survival and updated results for sunitinib compared with interferon alfa in patients with metastatic renal cell carcinoma. J Clin Oncol 2009;27:3584-90.

31. Motzer RJ, Escudier B, Tomczak P, Hutson TE, Michaelson MD, Negrier S, et al. Axitinib versus sorafenib as second-line treatment for advanced renal cell carcinoma: overall survival analysis and updated results from a randomised phase 3 trial. Lancet Oncol 2013;14:552-62.

32. Tan SS, Bouwmans CA, Rutten FF, Hakkaart-van Roijen L. Update of the Dutch Manual for Costing in Economic Evaluations. Int J Technol Assess Health Care 2012;28:152-8.

33. Nederlandse Zorgautoriteit. Tariffs as issued by the Dutch Healthcare Authority [in Dutch: DBC zorgproducten tariefapplicatie]. Available at: [http://dbc-zorgproducten-tarieven.nza.nl/nzaZpTarief. Last updated January 2015](http://dbc-zorgproducten-tarieven.nza.nl/nzaZpTarief.%20Last%20updated%20January%202015).
