# Supplementary material for: Potential health gains for patients with metastatic renal cell carcinoma in daily clinical practice: A real-world cost-effectiveness analysis of sequential first- and second-line treatments
Source: PLoS One. 2017 May 22;12(5):e0177364. doi: 10.1371/journal.pone.0177364 (PMC5439671; doi:10.1371/journal.pone.0177364)
Supplement: S1 Table — (DOCX) [file pone.0177364.s002.docx]

**Table S1. Utility weights**

| Prognosis | Utility  *Mean* | Source |
| --- | --- | --- |
| Favourable or intermediate prognosis at diagnosis | 0.725* | 17 |
| Poor prognosis at diagnosis | 0.590 | 18 |
| Favourable or intermediate prognosis before start second-line therapy | 0.700** | 19 |
| Poor prognosis before start second-line therapy | 0.590 | 18 |

* Average of utility at baseline for patients receiving pazopanib and patients receiving placebo ** Average of utility for patients treated with axitinib and patients treated with sorafenib.
