# Supplementary figures and images for: Potential health gains for patients with metastatic renal cell carcinoma in daily clinical practice: A real-world cost-effectiveness analysis of sequential first- and second-line treatments
Source: PLoS One. 2017 May 22;12(5):e0177364. doi: 10.1371/journal.pone.0177364 (PMC5439671; doi:10.1371/journal.pone.0177364)

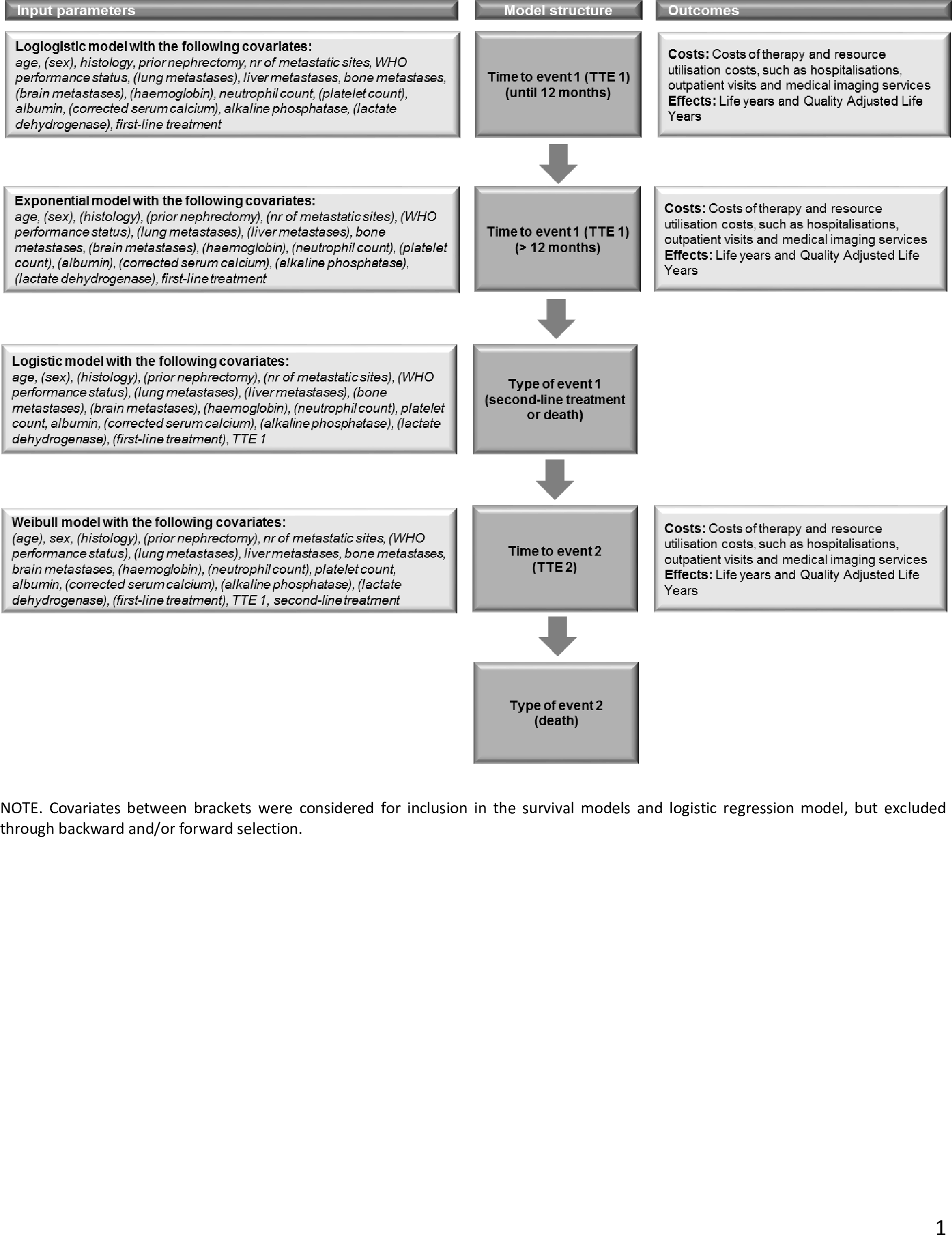

Supplement: S1 Fig — (TIF) [file pone.0177364.s003.tif]

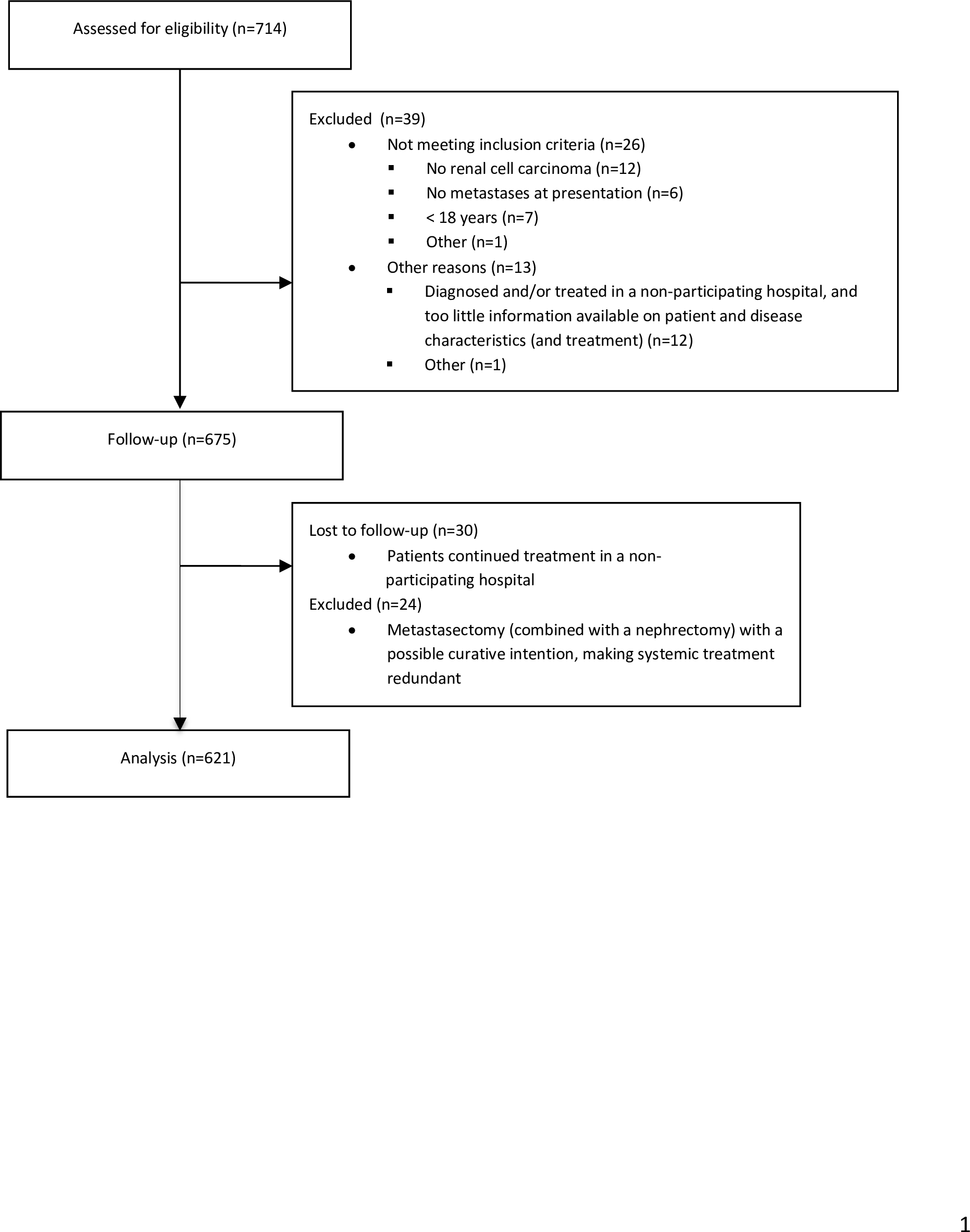

Supplement: S2 Fig — (TIF) [file pone.0177364.s004.tif]

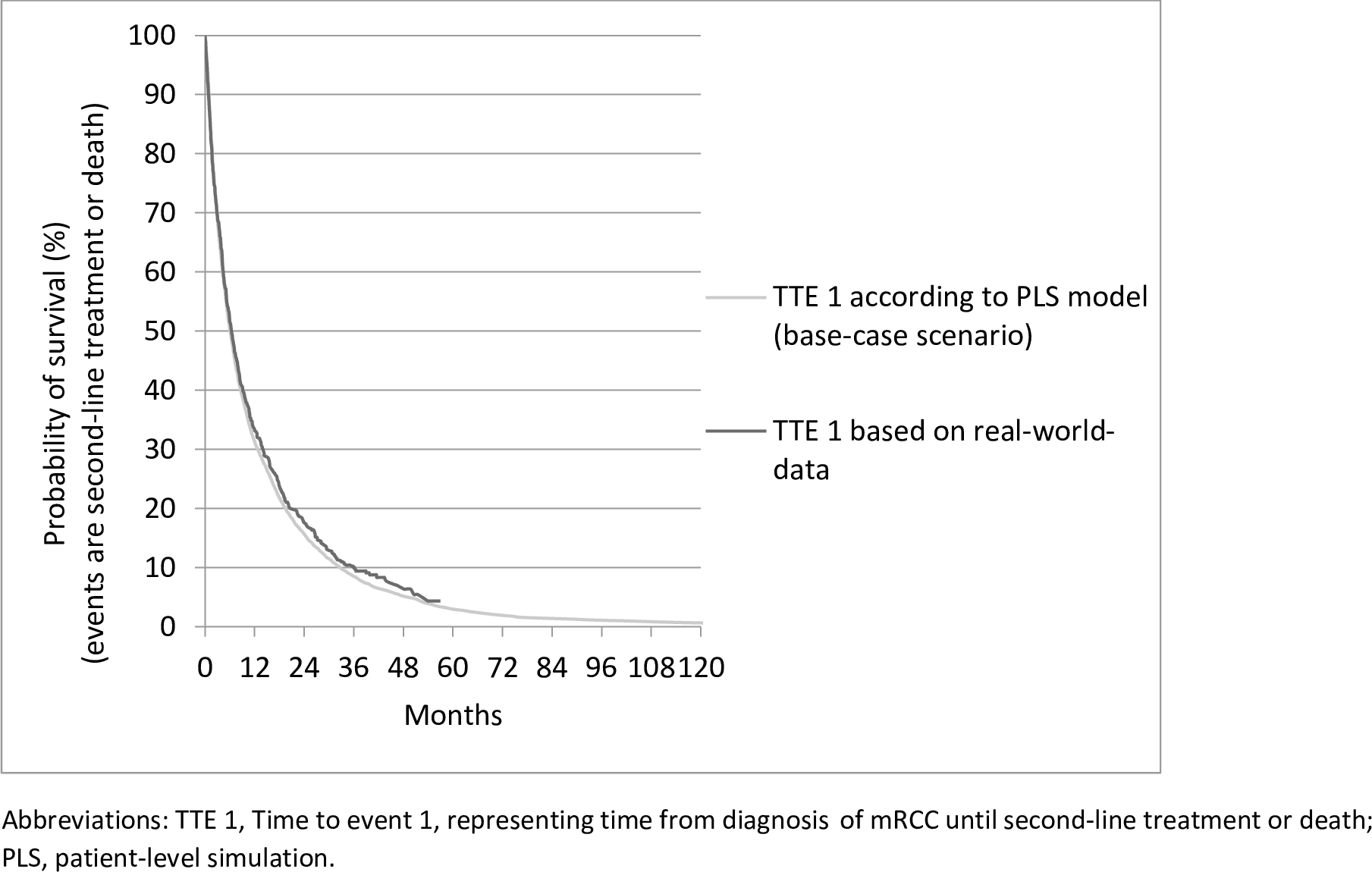

Supplement: S3 Fig — (TIF) [file pone.0177364.s005.tif]

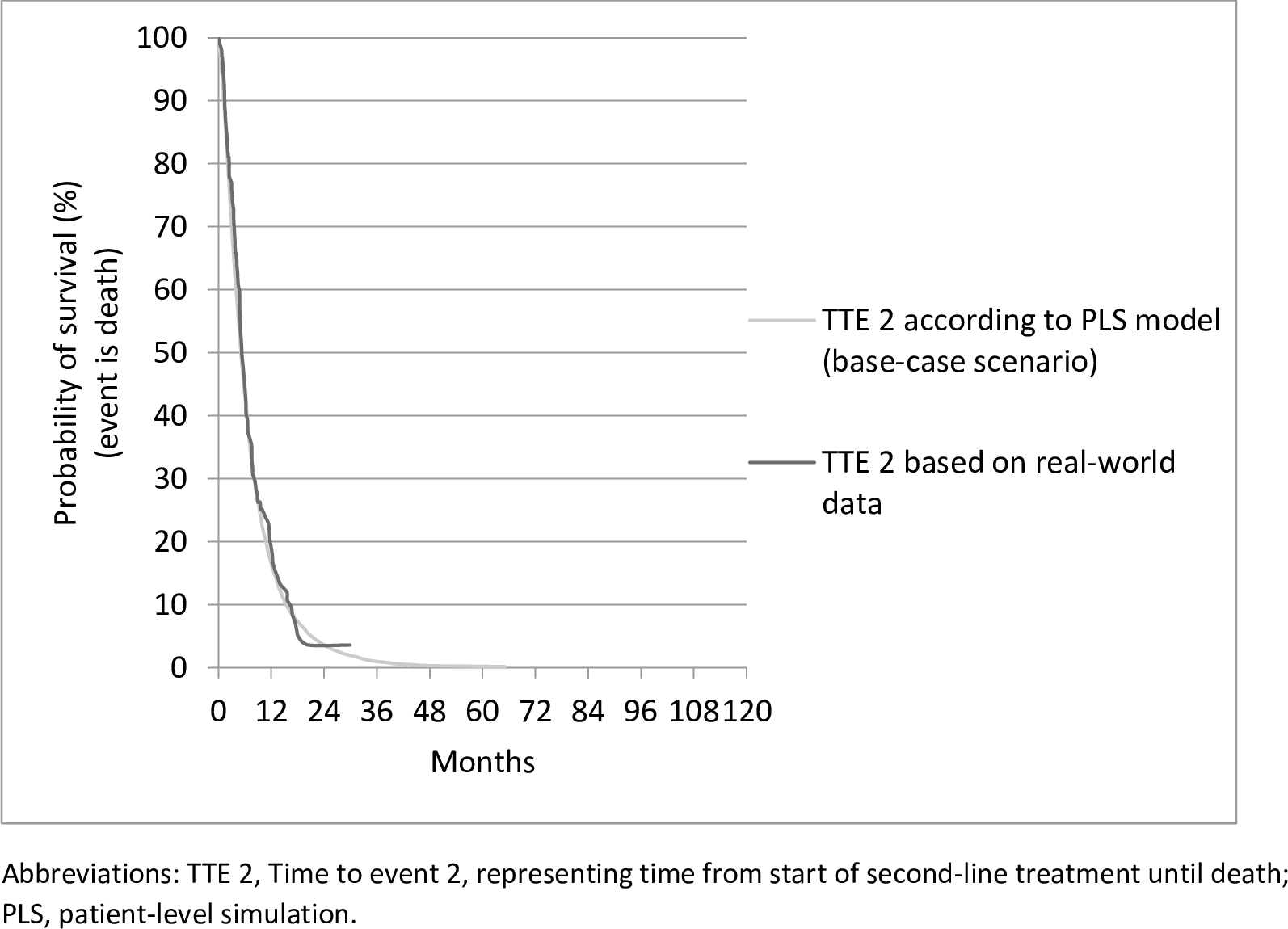

Supplement: S4 Fig — (TIF) [file pone.0177364.s006.tif]

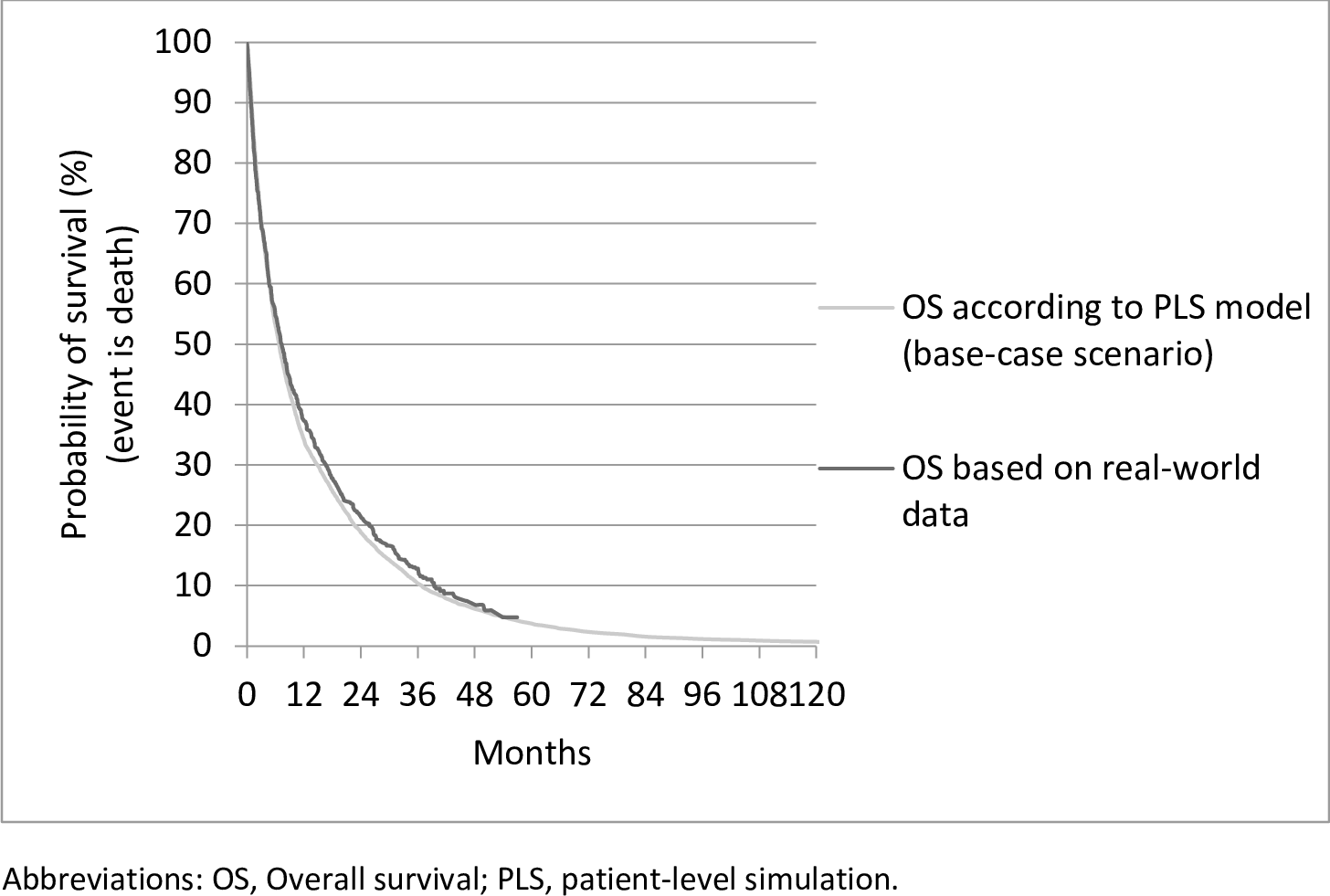

Supplement: S5 Fig — (TIF) [file pone.0177364.s007.tif]
